# Abnormal Liver Function Tests Were Related to Short- and Long-Term Prognosis in Critically Ill Patients With Primary Pulmonary Hypertension

**DOI:** 10.3389/fcvm.2022.897040

**Published:** 2022-06-02

**Authors:** Dayu Wang, Suiqing Huang, Guangtao Xu, Sha Wu, Zhen Liu, Long Xu, Bo Hu, Jian Hou

**Affiliations:** ^1^Department of Cardiology, Guangzhou Panyu Central Hospital, Guangzhou, China; ^2^Department of Cardiac Surgery, The First Affiliated Hospital of Sun Yat-sen University, Guangzhou, China; ^3^Forensic and Pathology Laboratory, Department of Pathology, Institute of Forensic Science, Jiaxing University, Jiaxing, China; ^4^Department of Pathology and Municipal Key-Innovative Discipline of Molecular Diagnostics, Jiaxing Hospital of Traditional Chinese Medicine, Jiaxing University, Jiaxing, China

**Keywords:** primary pulmonary hypertension, 90-day mortality, long-and short-term prognoses, hospital mortality, 4-year mortality, liver function tests, intensive care unit

## Abstract

**Aim:**

The aim of this study was to examine the utility of liver function tests (LFTs) in predicting the prognosis of critically ill patients with primary pulmonary hypertension (PPH) with/without liver disease.

**Methods:**

We retrieved the Medical Information Mart for Intensive Care III (MIMIC-III) database to acquire clinical data. From the database, we recruited adult patients that were equal to or older than 18 years with primary pulmonary hypertension (PPH) discharge from intensive care unit (ICU). Then, the relationship between LFTs and duration of hospitalization and ICU stays was examined based on the Spearman correlation. The chi-square assessment was conducted to examine the correlation between LFTs and death rates. Survival curves were plotted with the aid of the Kaplan-Meier technique, and the curves were subsequently compared utilizing the log-rank test. The LFTs were identified as independent predictive variables of death according to the results of multivariable logistic regression. The specificity and sensitivity for mortality were calculated utilizing receiver operating characteristic (ROC) curves and the area under the curve (AUC).

**Results:**

In total, 198 patients satisfying the inclusion criteria were recruited, among which there were 23 patients with liver disease. Only ALB was correlated with the length of ICU stay in the total PPH group. ALB independently served as a risk variable for hospital mortality and 90-day mortality and was significantly associated with 90-day and 4-year survival rates in both total PPH and PPH without liver disease. AST was correlated with hospital mortality and 90-day survival curves in both total PPH and PPH without liver disease and independently served as a risk factor for hospital and 90-day mortality only in the total PPH group. ALT independently acted as a risk variable for hospital mortality and total bilirubin was correlated with hospital mortality in the total group. The diagnostic performance of the predictive model combining the LFTs was moderately good for the hospital, 90-day, and 4-year mortality. Both Modelı End-Stage ıLiverı Disease (MELD) score and albumin-bilirubin (ALBI) score were independent risk factors for short- and long-term prognosis. And they were also significantly associated with short- and long-term prognosis.

**Conclusion:**

Among critically ill patients with PPH and with or without liver illness, aberrant LFT was linked to short- and long-term prognoses.

## Introduction

Primary pulmonary hypertension (PPH), one of the pulmonary arterial hypertension (PAH), is an uncommon chronic illness hallmarked by right ventricle (RV) failure and poor prognosis (1). In recent years, PAH has shown to be a multiorgan systemic disorder hallmarked by anomalies in the immune system, skeletal muscle, kidneys, peripheral and central nervous system, as well as systemic circulation (2, 3). Due to the liver’s close physiological and anatomical link to the RV, it is among the vulnerable organs to be affected by RV failure from PAH, which can lead to a variety of liver abnormalities (4).

The WHO functional class, average right atrial pressure, 6-min walking distance, and brain natriuretic peptide (BNP) plasma levels served as prognostic indicators for PAH in this research (5). Recently, abnormal liver function tests (LFTs) were indicated to significantly affect the prognosis of PAH. Decreased ALB was associated with decreased PAH survival (6, 7). Increased bilirubin level was correlated with an elevated mortality risk of PAH (8, 9). Another research report showed that it was not associated with PAH survival (10). The inconsistency of the current studies suggested the mechanistic complexity of LFTs in PAH. However, not only ischemic hepatitis and congestive hepatopathy caused by heart failure due to PAH but also preexisting liver disease could cause abnormal LFTs. Although the correlation between LFT abnormalities and PAH was recently reported, few investigations compared the abnormal liver markers caused by PAH with and without liver disease.

The objective of this research was to examine if there was a correlation between LFTs and duration of hospitalization and ICU stays, inpatient mortality, 90-day mortality, and 4-year mortality among critically ill PPH patients with or without liver illness.

## Materials and Methods

### Data Source

A retrospective cohort approach was adopted in this research. The ICU database, which is a public critical care repository of Medical Information Mart for Intensive Care III (MIMIC-III), was retrieved to obtain data. Beginning from 2001 to 2012, the clinical data from patients from ICU admissions at Beth Israel Deaconess Medical Center (BIDMC) were gathered (11). The access approval to the database was granted by the institutional review boards of both the BIDMC and the Massachusetts Institute of Technology Affiliates. Since the data had been de-identified, there was no need for informed consent.

### Patient Selection

The MIMIC-III database was utilized for the purpose of identifying clinical data from patients who met the criteria for inclusion in this research. The aforementioned criteria were (1) patients who have undergone a definite diagnosis of PPH. Diagnoses of PPH were made in accordance with the International Classification of Diseases, Ninth Revision, Clinical Modification (ICD-9-CM) codes. For PPH, the ICD-90-CM code was 4160. (2) Patients older than 18 years, which was reported previously (12). (3) Patients who underwent routine blood tests 24 h upon admission, and the tested indicators included white blood cell (WBC), serum sodium, serum potassium, platelets, blood urea nitrogen (BUN), creatinine, total bilirubin, blood alanine transaminase (ALT), blood aspartate transaminase (AST), and blood albumin (ALB). The criteria for exclusion used in this research were (1) patients who did not develop PPH; (2) patients under the age of 18; (3) patients whose blood test results were not complete; and (4) patients with PPH suffering from systemic immune system diseases, active infectious disease; other important functional organs severely damaged, malignant tumors, etc.

### Data Acquisition

The data were acquired and collected with the aid of the Structured Query Language (SQL), and the administration platform used was pgAdmin4 for PostgreSQL. The acquired data predominantly consist of demographics (gender and age), vital signs [temperature, percutaneous oxygen saturation (SpO_2_), respiratory rate (RR), systolic blood pressure (SBP), heart rate (HR), and diastolic blood pressure (DBP)], comorbid conditions (renal failure, liver disease, complicated diabetes, uncomplicated diabetes, chronic pulmonary, hypertension, peripheral vascular, pulmonary circulation disorder, congestive heart failure, valvular illness, and cardiac arrhythmias), laboratory tests (serum potassium, platelets, BUN, WBC, creatinine, ALB, total bilirubin, AST, ALT, and serum sodium), Model End-Stage Liver Disease (MELD), the Sequential Organ Failure Assessment (SOFA) score, and the Simplified Acute Physiology Score (SAPS) II. ALBI score was calculated using albumin and total bilirubin, and FIB-4 index was calculated using age, ALT, AST, and platelet by formula as described before (13, 14). The formulae for calculating the scores are MELD score = 3.78 × ln [(bilirubin (mg/dl)] + 11.2 × ln (INR) + 9.57 × ln [creatinine (mg/dl) + 6.43 × (for cholestatic or alcoholic liver disease and 1 for others)]; ALBI score = [log_10_ bilirubin (μmol/L) × 0.66] + [albumin (g/L) × −0.085]; FIB-4 index = [age (years) × AST (U/L)]/[Platelet count (10^9^/L) × ALT (U/L)^1/2^]. ALBI scores were divided into three levels, namely, grade 1, ALBI ≤ −2.60; grade 2, −2.60 < ALBI ≤ −1.39; grade 3: ALBI > −1.39. MELD scores were divided into three levels: grade 1: MELD ≤ 14; grade 2: 14 < MELD ≤ 18; and grade 3: MELD > 18. As the percentage of incomplete information for each variable was less than 1.5%, it is reasonable to ignore these variables in subsequent analyses.

### Outcome Variables

The outcome variables that were obtained in this research are hospital mortality, duration of ICU stay, duration of hospitalization stay, 90-day mortality (post-ICU admission), as well as 4-year mortality. As a patient might undergo ICU admissions several times throughout a single hospitalization, the time spent in the ICU was exclusively determined by the first ICU hospitalization. Only individuals in the CareVue system who had been monitored for no less than 4 years were included in the analysis for 4-year mortality.

### Statistical Analysis

Categorical data are expressed as numerals with percentages and were examined by performing the χ^2^ test. Continuous variables are reported as mean ± standard deviation or median (interquartile range) and were subjected to a comparison by performing the Mann–Whitney *U*-test and *t*-test. The non-parametric Spearman’s rank correlation test was conducted for the purpose of assessing the correlation between the duration of ICU and hospital stays as well as the lab measurements. We included all patients with PPH, containing survivors and non-survivors, in the prognostic analysis. Survival curves were plotted with the help of the Kaplan–Meier technique and subjected to a subsequent comparison utilizing the log-rank test. We performed multivariate and univariate logistic regression analyses to screen for independent predictive indicators for PPH-related mortality (including hospital, 90-day, and 4-year mortality). To account for possible confounding factors, two varied models were developed. For total patients with PPH, model 1 was corrected for BUN, valvular disease, SOFA scores, liver disease, SpO_2_, and RR, whereas model 2 was adjusted for SAPS II score and valvular disease. For PAH patients without liver-related disorders, model 1 was corrected for BUN, SOFA score, SpO_2_, valvular disorder, and MELD score, whereas model 2 was corrected for SAPS II score and valvular disease. Statistical significance was set as a *p*-value < 0.05. Additionally, the specificity and sensitivity were assessed with the aid of receiver operating characteristic (ROC) curves and the area under the curve (AUC) values. STATA, version 14.0 (StataCorp, College Station, TX, United States) was employed to perform all the analyses of statistical data.

## Results

### The Study Population’s Baseline Parameters

In total, 198 patients who satisfied the inclusion requirements were included in this research, with 26 patients (13.13%) dying in the hospital. Table 1 summarizes the baseline features of the included patients, including demographics, lab tests, vital signs, comorbid conditions, and scores.

**TABLE 1:** Baseline characteristics of the study population with different survival status in hospital.

	Survivors (*n* = 172)	Non-survivors (*n* = 26)	*P*-value
**Demographics**			
Age	70.04 ± 14.43	67.37 ± 15.18	0.384
Male, *n* (%)	79(45.93%)	14(53.85%)	0.451
Weight (kg)	81.0(66.0–93.36)	85.96 (66–103)	0.271
Height (cm)	167.64 (160.02–177.8)	162.56 (158.12–168.91)	0.072
**Vital signs**			
HR, beats/minute	82.17 (73.87–91.74)	86.53 (74.2–99.96)	0.059
SBP, mmHg	114.25 (104.58–125.36)	105.85 (94.75–118.47)	0.010
DBP, mmHg	55.51 (50.01–62.67)	59.64(49.98–66.67)	0.281
RR, times/minute	18.6 (16.33–20.71)	21.20 (17.61–23.52)	0.002
Temperature, °C	36.76 (36.40–37.08)	36.46 (36.01–37.46)	0.017
SpO_2_, %	97.42 (96.07–98.64)	96.19 (94.92–97.83)	0.004
**Laboratory events**			
WBC, 10^9^/L	9.3 (6.8–13.5)	9.7 (7.9–15.6)	0.470
Serum sodium, mmol/L	139 (136–141)	138 (137–141)	0.828
Serum potassium, mmol/L	4.2 (3.8–4.7)	4 (3.8–4.4)	0.119
Glucose, mg/dL	131.6 (114.8–156.0)	133.92 (117–188)	0.352
Platelets, 10^9^/L	201 (142–266)	195.5 (125–263)	0.474
BUN, mg/dL	25 (16–40)	30.5 (23–59)	0.023
Creatinine, mg/dL	1.1 (0.9–1.6)	1.6 (1.1–2.0)	0.260
ALB, g/dL	3.5 (3.1–3.9)	3.0 (2.7–3.5)	0.000
ALT, IU/L	21 (14.5–35.5)	29.5 (20–64)	0.000
AST, IU/L	25.5 (19–45.5)	52.5 (28–122)	0.000
Total bilirubin, mg/dL	0.6 (0.4–1.0)	0.65(0.3–1.2)	0.017
**Comorbidities**			
Congestive heart failure	110(63.95%)	14 (53.85%)	0.321
Cardiac arrhythmias	86 (50.00%)	11 (42.31%)	0.465
Valvular disease	56 (32.56%)	2 (7.69%)	0.009
Pulmonary circulation disorder	166 (96.51%)	26 (100.0%)	0.333
Peripheral vascular	14 (8.14%)	2 (7.69%)	0.938
Hypertension	91 (52.91%)	11 (42.31%)	0.313
Chronic pulmonary	36 (20.93%)	8 (30.77%)	0.261
Uncomplicated diabetes	43 (25.00%)	3 (11.54%)	0.130
Complicated diabetes	11 (6.40%)	1 (3.85%)	0.612
Liver disease	14 (8.14%)	9 (34.62%)	<0.001
Renal failure	33 (19.190%)	5 (19.23%)	0.996
**Scores**			
SAPS II	36 (26–44.5)	47.5 (42–56)	<0.001
SOFA	5 (3–7)	7 (4–10)	<0.001
MELD	15 (10–19.31)	19 (16–26.16)	0.000
FIB-4	2.12 (1.41–3.61)	4.92 (1.76–6.77)	0.158
ALBI	−1.98 (−2.30 – −1.63)	−1.40 (−1.97 – −1.29)	<0.001

*Values are presented as the mean ± standard deviation, median (interquartile range), or number of patients (%). ALB, blood albumin; ALT, blood alanine transaminase; AST, blood aspartate transaminase; BUN, blood urea nitrogen; HR, heart rate; SBP, systolic blood pressure; DBP, diastolic blood pressure; RR, respiratory rate; SpO_2_, percutaneous oxygen saturation; WBC, white blood cell; SAPS II, Simplified Acute Physiology Score II; SOFA, Sequential Organ Failure Assessment; MELD, Model End-Stage Liver Disease.*

Table 1 displays the demographic data from the research sample. Among the survivors and non-survivors, no remarkable differences were found in age, gender, weight, or height. Non-survivors exhibited remarkably lowered ALB levels (3.5 vs. 3.0, *p* < 0.001) and elevated levels of total bilirubin (0.6 vs. 0.65, *p* < 0.001), AST (25.5 vs. 52.5, *p* < 0.001), ALT (21 vs. 29.5, *p* < 0.001), MELD score (15 vs. 19, *p* < 0.001), and ALBI score (−1.98 vs. −1.40, *p* < 0.001) (Table 1). Non-survivors exhibited decreased SpO_2_, temperature, SBP levels and incidence of valvular disorder, as well as elevated BUN, RR, SOFA, SAPS II, and MELD scores, and also experienced a history of liver (Table 1).

### The Liver Function Index and Its Prognostic Value in Primary Pulmonary Hypertension

Table 2 illustrates a correlation between the liver function index and the duration of hospitalization and ICU stays among patients with PPH (total patients with PPH or patients with PPH who do not have liver illness), which was investigated by performing Spearman’s rank correlation analysis. Only ALB exhibited a remarkable negative correlation with the duration of ICU stay for total patients with PPH (Spearman’s rho = −0.142, *p* = 0.046).

**TABLE 2:** The correlation of ALB, ALT, AST, and total bilirubin with hospital stay and ICU stay.

		Length of Hospital stay		Length of ICU stay	
		Total	Without liver disease	Total	Without liver disease
ALB, g/dL	Spearman’s Rho	–0.101	–0.103	**−−0.142**	–0.131
	*P*-value	0.155	0.173	**0.046**	0.083
ALT, IU/L	Spearman’s Rho	0.010	–0.010	–0.001	–0.073
	*P*-value	0.886	0.895	0.988	0.335
AST, IU/L	Spearman’s Rho	–0.005	–0.030	–0.071	–0.166
	*P*-value	0.943	0.692	0.321	0.028
Total bilirubin, mg/dL	Spearman’s Rho	–0.032	–0.019	0.026	–0.014
	*P*-value ho	0.655	0.806	0.716	0.854

*The p-values of less than 0.05 are indicated in bold.*

Then, we probed into the relationship between liver function index and hospital mortality among patients with PPH (total patients with PPH or patients with PPH who do not have liver illness). A remarkable correlation was discovered between quartiles of ALB and AST and hospital mortality in total patients with PPH and patients with PPH and without liver disease (for total patients with PPH, ALB: *p* = 0.006; AST: *p* = 0.012; for patients with PPH and without liver disease, ALB: *p* = 0.023; AST: *p* = 0.042) (Table 3). In both total PPH and PPH without liver disease groups, a greater incidence of hospital mortality was reported during the 1st quartile for ALB, whereas a greater proportion of hospital mortality was observed within the 4th quartile for AST. In the case where clinical normal values were grouped together, similar tendencies emerged. In both total PPH and PPH without liver disease groups, a greater incidence of hospital mortality was found among the group exhibiting a lesser value compared to normal for ALB, whereas in the total PPH group, a greater proportion of patients experiencing hospital mortality was reported among the group with a greater level in contrast with normal for AST (for total patients with PPH, ALB: *p* = 0.008; AST: *p* = 0.003; for patients with PPH and without liver disease, ALB: *p* = 0.013) (Table 3). Within the total PPH group, patients with a greater value in total bilirubin exhibited an elevated incidence of hospital mortality (*p* = 0.035).

**TABLE 3:** The relationship between ALB, ALT, AST, and total bilirubin with hospital mortality.

Total PPH patients										PPH patients without liver disease								
	Q1	Q2	Q3	Q4	*P*-value	Lower	Normal	Higher	*P*-value	Q1	Q2	Q3	Q4	*P*-value	Lower	Normal	Higher	*P*-value
**ALB**																		
Survivors	53 (75.71%)	40 (90.91%)	34 (91.89%)	45 (95.74%)	**0.006**	78 (80.41%)	94 (98.55%)	0 (0%)	**0.008**	47 (81.03%)	38 (95.00%)	29 (90.63%)	44 (97.78%)	**0.023**	71 (84.52%)	87 (95.60%)	0 (0%)	**0.013**
Non-survivors	17 (24.29%)	4 (9.09%)	3 (8.11%)	2 (4.26%)		19 (19.59%)	7 (1.45%)	0 (0%)		11 (18.97%)	2 (5.00%)	3 (9.38%)	1 (2.22%)		13 (15.48%)	4 (4.40%)	0 (0%)	
**ALT**																		
Survivors	59 (93.65%)	39 (88.64%)	35 (81.40%)	39 (81.25%)	0.163	2 (100.00%)	134 (88.74%)	36 (80.00%)	0.269	56 (94.92%)	35 (92.11%)	31 (81.58%)	36 (77.50%)	0.181	1 (100.00%)	124 (90.51%)	33 (89.19%)	0.920
Non-survivors	4 (6.35%)	5 (11.36%)	8 (18.60%)	9 (18.75%)		0 (0%)	17 (11.26%)	9 (20.00%)		3 (5.08%)	3 (7.89%)	7 (18.42%)	4 (22.50%)		0 (0%)	13 (9.49%)	4 (10.81%)	
**AST**																		
Survivors	57 (89.06%)	42 (97.67%)	38 (86.36%)	35 (74.47%)	**0.012**	0 (0.00%)	123 (91.79%)	49 (76.56%)	**0.003**	54 (90.00%)	42 (100.00%)	32 (88.89%)	30 (81.08%)	**0.042**	0 (0.00%)	117 (92.13%)	41 (85.42%)	0.181
Non-survivors	7 (10.94%)	1 (2.33%)	6 (13.64%)	12 (25.53%)		0 (0.00%)	11 (8.21%)	15 (23.44%)		6 (10.00%)	0 (0.00%)	4 (11.11%)	7 (18.92%)		0 (0.00%)	10 (7.87%)	7 (14.58%)	
**Total bilirubin**																		
Survivors	85 (85.00%)	22 (100.00%)	30 (88.24%)	35 (83.33%)	0.245	32 (88.89%)	134 (88.16%)	6 (60.00%)	**0.035**	83 (88.30%)	20 (100.00%)	28 (87.50%)	27 (93.10%)	0.373	30 (90.91%)	122 (90.37%)	6 (85.71%)	0.913
Non-survivors	15 (15.00%)	0 (0.00%)	4 (11.76%)	7 (16.67%)		4 (11.11%)	18 (11.84%)	4 (40.00%)		11 (11.70%)	0 (0.00%)	4 (12.50%)	2 (6.90%)		3 (9.09%)	13 (9.63%)	1 (14.29%)	

*Q, quartile of serum ALB, ALT, AST, and total bilirubin value; Lower, lower than clinical normal value; Normal, clinical normal value; Higher, higher than clinical normal value. The p-values of less than 0.05 are indicated in bold.*

The relationship between the liver function index and PPH mortality over 90 days was also studied, with the findings reported in Table 4. For ALB, a higher incidence rate of 90-day mortality was found in the 1st quartile. In addition, for AST, a higher incidence rate of 90-day mortality was found in the 4th quartile in the total PPH group but not PPH without liver disease (ALB: *p* = 0.025; AST: *p* = 0.031). In the case where clinical normal values were grouped together, comparable tendencies emerged. For ALB, in both total PPH and PPH without liver disease groups, the group exhibiting a lesser value compared to the normal had a greater risk of 90-day mortality. In addition, for AST, a higher incidence rate of hospital mortality was found in patients belonging to the group with a higher value than normal only in the total PPH group (for total patients with PPH, ALB: *p* = 0.001; AST: *p* = 0.022; for patients with PPH and without liver disease, ALB: *p* = 0.001) (Table 4).

**TABLE 4:** The relationship between ALB, ALT, AST, and total bilirubin with 90-day mortality.

Total PPH patients										PPH patients without liver disease								
	Q1	Q2	Q3	Q4	*P*-value	Lower	Normal	Higher	*P*-value	Q1	Q2	Q3	Q4	*P*-value	Lower	Normal	Higher	*P*-value
**ALB**																		
Survivors	49 (70.00%)	34 (77.27%)	32 (86.49%)	43 (91.49%)	**0.025**	68 (70.10%)	90 (89.11%)	0 (0%)	**0.001**	43 (74.14%)	32 (80.00%)	28 (87.50%)	42 (93.33%)	0.063	61 (72.62%)	84 (92.31%)	0 (0%)	**0.001**
Non-survivors	21 (30.00%)	10 (22.73%)	5 (13.51%)	4 (8.51%)		29 (29.90%)	11 (10.89%)	0 (0%)		15 (25.86%)	8 (20.00%)	4 (12.50%)	3 (6.67%)		23 (27.38%)	7 (7.69%)	0 (0%)	
**ALT**																		
Survivors	51 (80.95%)	37 (84.09%)	34 (79. 07%)	36 (75.00%)	0.740	1 (50.00%)	124 (82.12%)	33 (73.33%)	0.250	49 (83.05%)	33 (86.84%)	30 (78.95%)	33 (82.50%)	0.840	1 (100.00%)	114 (83.21%)	30 (81.08%)	0.860
Non-survivors	12 (19.05%)	7 (15.91%)	9 (20.93%)	12 (25.00%)		1 (50.00%)	27 (17.88%)	12 (26.67%)		10 (16.95%)	5 (13.16%)	8 (21.05%)	7 (17.50%)		0 (0%)	23 (16.79%)	7 (18.92%)	
**AST**																		
Survivors	50 (78.13%)	40 (93.02%)	36 (81.82%)	32 (68.09%)	**0.031**	0 (0.00%)	113 (84.33%)	45 (70.31%)	**0.022**	48 (80.00%)	40 (95.24%)	30 (83.33%)	27 (72.97%)	0.059	0 (0.00%)	108 (85.04%)	37 (77.08%)	0.213
Non-survivors	14 (21.88%)	3 (6.98%)	8 (18.18%)	15 (31.91%)		0 (0.00%)	21 (15.67%)	19 (29.69%)		12 (20.00%)	2 (4.76%)	6 (16.67%)	10 (27.03%)		0 (0.00%)	19 (14.96%)	11 (22.92%)	
**Total bilirubin**																		
Survivors	79 (79.00%)	21 (95.45%)	26 (76.47%)	32 (76.19%)	0.266	31 (86.11%)	121 (79.61%)	6 (60.00%)	0.190	77 (81.91%)	19 (95.00%)	24 (75.00%)	25 (86.20%)	0.289	29 (87.88%)	110 (81.48%)	6 (85.71%)	0.668
Non-survivors	21 (21.00%)	1 (4.55%)	8 (23.53%)	10 (23.81%)		5 (13.89%)	31 (20.39%)	4 (40.00%)		17 (18.09%)	1 (5.00%)	8 (25.00%)	4 (13.79%)		4 (12.12%)	25 (18.52%)	1 (14.29%)	

*Q, quartile of serum ALB, ALT, AST, and total bilirubin value; Lower, lower than clinical normal value; Normal, clinical normal value; Higher, higher than clinical normal value. The p-values of less than 0.05 are indicated in bold.*

Only participants in CareVue who underwent a follow-up for no less than 4 years were examined for 4-year mortality. According to the results, no considerable association was discovered between the liver function index and PPH-related 4-year mortality, as indicated in Table 5.

**TABLE 5:** The relationship between ALB, ALT, AST, and total bilirubin with 4-year mortality.

Total PPH patients										PPH patients without liver disease								
	Q1	Q2	Q3	Q4	*P*-value	Lower	Normal	Higher	*P*-value	Q1	Q2	Q3	Q4	*P*-value	Lower	Normal	Higher	*P*-value
**ALB**																		
Survivors	27 (51.92%)	12 (32.43%)	13 (44.83%)	22 (61.11%)	0.089	32 (41.56%)	42 (54.55%)	0 (0%)	0.107	23 (53.49%)	12 (33.33%)	13 (50.00%)	21 (61.74%)	0.108	29 (42.03%)	40 (57.14%)	0 (0%)	0.075
Non-survivors	25 (48.08%)	25 (67.57%)	16 (55.17%)	14 (38.89%)		45 (58.44%)	35 (45.45%)	0 (0%)		20 (46.51%)	24 (66.67%)	13 (50.00%)	13 (38.24%)		40 (57.97%)	30 (42.86%)	0 (0%)	
**ALT**																		
Survivors	22 (43.14%)	20 (58.82%)	13 (40. 63%)	19 (51.035%)	0.401	0 (0.00%)	55 (47.01%)	19 (54.29%)	0.294	21 (44.68%)	18 (62.07%)	12 (40.00%)	18 (54.55%)	0.297	0 (0.00%)	51 (47.66%)	18 (58.06%)	0.362
Non-survivors	29 (56.86%)	14 (41.18%)	19 (59.38%)	18 (48.65%)		2 (100.00%)	62 (52.99%)	16 (45.71%)		26 (55.32%)	11 (37.97%)	18 (60.00%)	15 (45.45%)		1 (100.0%)	56 (52.34%)	13 (41.94%)	
**AST**																		
Survivors	24 (50.00%)	22 (61.11%)	13 (38.24%)	15 (41.67%)	0.218	0 (0.00%)	54 (51.43%)	20 (40.82%)	0.220	23 (52.27%)	22 (62.86%)	11 (36.67%)	13 (43.33%)	0.167	0 (0.00%)	52 (52.53%)	17 (42.50%)	0.285
Non-survivors	24 (40.00%)	14 (38.89%)	21 (61.76%)	21 (58.33%)		0 (0.00%)	51 (48.57%)	29 (59.18%)		21 (47.73%)	13 (37.14%)	19 (63.33%)	17 (56.67%)		0 (0.00%)	47 (47.47%)	23 (57.50%)	
**Total bilirubin**																		
Survivors	33 (44.59%)	12 (63.16%)	13 (44.83%)	16 (50.00%)	0.520	17 (53.13%)	56 (48.70%)	1 (14.29%)	0.170	33 (46.48%)	12 (66.67%)	12 (42.86%)	12 (54.55%)	0.377	16 (55.17%)	52 (49.52%)	1 (20.00%)	0.348
Non-survivors	41 (55.41%)	7 (36.84%)	16 (55.17%)	16 (50.00%)		15 (46.88%)	59 (51.30%)	6 (85.71%)		38 (53.52%)	6 (33.33%)	16 (57.14%)	10 (45.45%)		13 (44.83%)	53 (50.48%)	4 (80.00%)	

*Q, quartile of serum ALB, ALT, AST, and total bilirubin value; Lower, lower than clinical normal value; Normal, clinical normal value; Higher, higher than clinical normal value. The p-values of less than 0.05 are indicated in bold.*

**FIGURE 1:**
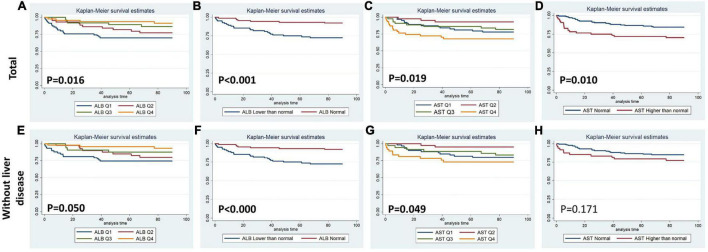
Kaplan–Meier 90-day survival curves comparing patients by liver function index levels. Q, quartile of serum ALB, ALT, AST, and total bilirubin value; normal, clinical normal value; ALB, blood albumin; ALT, blood alanine transaminase; AST, blood aspartate transaminase. **(A–D)** Total patients with PPH. **(E–H)** Patients with PPH and without liver disease.

**FIGURE 2:**
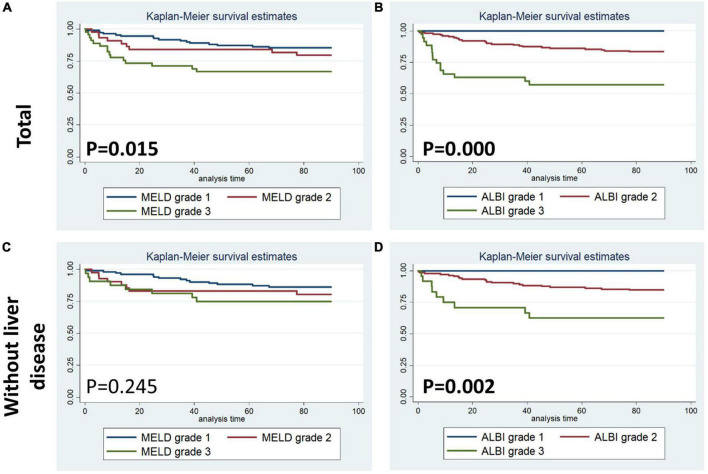
Kaplan–Meier 90-day survival curves comparing patients by liver function score levels (MELD and ALBI score). **(A,B)** Total patients with PPH. **(C,D)** Patients with PPH and without liver disease.

**FIGURE 3:**
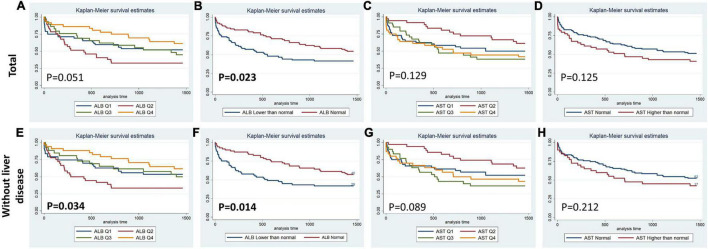
Kaplan–Meier 4-year survival curves comparing patients by liver function index levels. Q, quartile of serum ALB, ALT, AST, and total bilirubin value; Normal, clinical normal value; ALB, blood albumin; ALT, blood alanine transaminase; AST, blood aspartate transaminase. **(A–D)** Total patients with PPH. **(E–H)** Patients with PPH and without liver disease.

**FIGURE 4:**
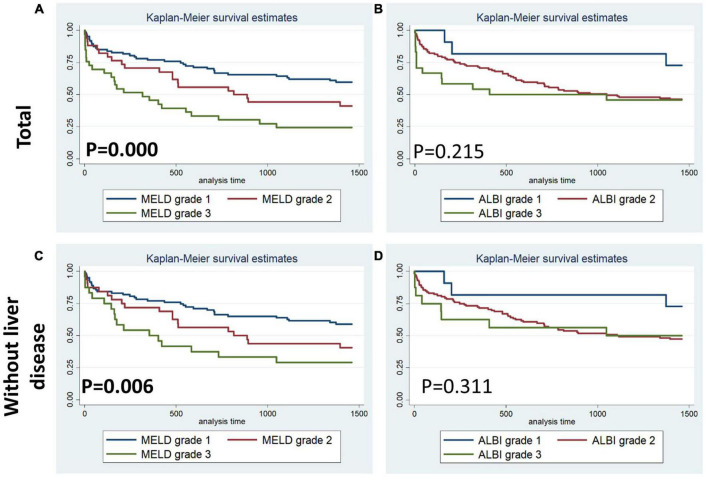
Kaplan–Meier 4-year survival curves comparing patients by liver function score levels (MELD and ALBI score). **(A,B)** Total patients with PPH. **(C,D)** Patients with PPH and without liver disease.

Figures 1–4 display Kaplan–Meier survival curves comparing patients based on their liver function indices and liver function score levels. As depicted by Figure 1, patients within the first and fourth quartile of ALB and AST, respectively, exhibited the worst 90-day survival in both total PPH and PPH without liver disease groups, while patients with lower ALB than normal in both total PPH and PPH without liver disease groups, and higher AST than normal in total PPH group exhibited a reduced 90-day survival rate (all *p* < 0.05) (Figures 1A–F). As depicted in Figure 2, patients within grade 3 of MELD score exhibited the worst 90-day survival in both total PPH groups, while patients within grade 3 of ALBI score, respectively, exhibited the worst 90-day survival in both total PPH and PPH without liver disease groups (all *p* < 0.05) (Figures 2A–D). As displayed in Figure 3, patients within the fourth quartile of ALB had the highest 4-year survival in PPH without liver disease group, while patients with lower ALB than normal in both total PPH and PPH without liver disease groups had reduced 4-year survival rate (all *p* < 0.05) (Figures 3A–F). As displayed in Figure 4, patients within grade 3 of MELD score had the worst 4-year survival in both total PPH and PPH without liver disease groups (all *p* < 0.01); however, there are no differences among the 4-year survival of the three grades of ALBI score (Figures 4A–D).

Table 6 presents the findings of the univariate logistic regression analysis. Accordingly, ALB was correlated with hospital mortality (total: OR = 0.29, 95% CI = 0.16–0.62, *p* = 0.001; without liver disease: OR = 0.22, 95% CI = 0.08–0.57, *p* = 0.002) and 90-day mortality (total: OR = 0.33, 95% CI = 0.17–0.62, *p* = 0.001; without liver disease: OR = 0.26, 95% CI = 0.12–0.56, *p* = 0.001) in both total PPH and PPH without liver disease groups. ALT and AST were correlated with hospital mortality and 90-day mortality in total patients with PPH (For ALT, hospital mortality: OR = 1.00, 95% CI = 1.00–1.01, *p* = 0.041, 90-day mortality: OR = 1.00, 95% CI = 1.00–1.01, *p* = 0.049; for AST, hospital mortality: OR = 1.00, 95% CI = 1.00–1.01, *p* = 0.008, 90-day mortality: OR = 1.00, 95% CI = 1.00–1.01, *p* = 0.022). In the total PPH group, total bilirubin was correlated with hospital mortality (OR = 1.08, 95% CI = 1.00–1.16, *p* = 0.048). MELD score was correlated with hospital mortality (total: OR = 1.12, 95% CI = 1.06–1.18, *p* < 0.001), 90-day mortality (total: OR = 1.09, 95% CI = 1.04–1.14, *p* = 0.001) and 4-year mortality (total: OR = 1.09, 95% CI = 1.03–1.16, *p* = 0.003) in total PPH, and 4-year mortality (total: OR = 1.09, 95% CI = 1.02–1.17, *p* = 0.008) in PPH without liver disease groups. ALBI score was correlated with hospital mortality (total: OR = 3.38, 95% CI = 1.66–6.88, *p* = 0.001; without liver disease: OR = 3.49, 95% CI = 1.37–8.89, *p* = 0.009) and 90-day mortality (total: OR = 3.15, 95% CI = 1.68–5.92, *p* < 0.001; without liver disease: OR = 3.60, 95% CI = 1.66–7.84, *p* = 0.001) in both total PPH and PPH without liver disease groups.

**TABLE 6:** Univariate logistic regression analyses for prognosis in patients with PPH.

	Total PPH patients						Without liver disease					
	Hospital mortality		90-day mortality		4-year mortality		Hospital mortality		90-day mortality		4-year mortality	
Variable	OR (95% CI)	*P*	OR (95% CI)	*P*	OR (95% CI)	*P*	OR (95% CI)	*P*	OR (95% CI)	*P*	OR (95% CI)	*P*
ALB, g/dL	0.29 (0.16–0.62)	**0.001**	0.33 (0.17–0.62)	**0.001**	0.71 (0.40–1.28)	0.252	0.22 (0.08–0.57)	**0.002**	0.26 (0.12–0.56)	**0.001**	0.65 (0.34–1.24)	0.196
ALT, IU/L	1.00 (1.00–1.01)	**0.041**	1.00 (1.00–1.01)	**0.049**	1.00 (0.99–1.00)	0.405	1.00 (1.00–1.01)	0.788	1.00 (1.00–1.01)	0.568	1.00 (0.99–1.00)	0.732
AST, IU/L	1.00 (1.00–1.01)	**0.008**	1.00 (1.00–1.01)	**0.022**	1.00 (0.99–1.00)	0.170	1.00 (1.00–1.01)	0.210	1.00 (1.00–1.01)	0.232	1.00 (0.99–1.00)	0.533
Total bilirubin, mg/dL	1.08 (1.00–1.16)	**0.048**	1.06 (1.00–1.14)	0.096	1.04 (0.96–1.14)	0.328	0.87 (0.47–1. 62)	0.660	1.01 (0.82–1.25)	0.897	1.27 (0.82–2.00)	0.278
MELD	1.12 (1.06–1.18)	**0.000**	1.09 (1.04–1.14)	**0.001**	1.09 (1.03–1.16)	**0.003**	1.08 (1.00–1.17)	0.062	1.06 (1.00–1.14)	0.067	1.09 (1.02–1.17)	**0.008**
ALBI	3.38 (1.66–6.88)	**0.001**	3.15(1.68–5.92)	**<0.001**	1.45 (0.81–2.60)	0.207	3.49 (1.37–8.89)	**0.009**	3.60 (1.66–7.84)	**0.001**	1.57 (0.80–3.07)	0.191

*For 4-year mortality, only patients in the CareVue system who were followed up for at least 4 years were analyzed. The p-values of less than 0.05 are indicated in bold.*

Table 7 presents the outcomes of the multivariate analysis. For performing multivariate analyses, model 1 was corrected for SOFA scores, BUN, valvular illness, liver illness, SpO_2_, and RR, whereas model 2 was corrected for SAPS II score and valvular disease for total patients with PPH. For patients with PPH and without liver disease, model 1 was corrected for BUN, SpO_2_, SOFA scores, valvular disorder, and MELD score, whereas model 2 was corrected for SAPS II score and valvular disease. ALB was correlated with hospital stay and 90-day mortality in both total PPH and PPH without liver disease groups (hospital mortality for total PPH group, model 1: OR = 0.38, 95% CI = 0.16–0.92, *p* = 0.027, model 2: OR = 0.39, 95% CI = 0.17–0.91, *p* = 0.028; hospital mortality for PPH without liver disease group, model 1: OR = 0.27, 95% CI = 0.10–0.76, *p* = 0.015, model 2: OR = 0.32, 95% CI = 0.12–0.88, *p* = 0.027; 90-day mortality for total PPH group, model 1: OR = 0.40, 95% CI = 0.20–0.79, *p* = 0.008, model 2: OR = 0.40, 95% CI = 0.20–0.79, *p* = 0.009; 90-day mortality for PPH without liver disease group, model 1: OR = 0.28, 95% CI = 0.12–0.62, *p* = 0.002, model 2: OR = 0.33, 95% CI = 0.15–0.73, *p* = 0.006). ALT and AST were correlated with hospital mortality within the total PPH group (ALT: model 2, OR = 1.00, 95% CI = 1.00–1.01, *p* = 0.036; AST, model 1: OR = 1.00, 95% CI = 1.00–1.01, *p* = 0.029, model 2: OR = 1.00, 95% CI = 1.00–1.01, *p* = 0.003). AST was correlated with 90-day mortality in the total PPH group (model 2, OR = 1.00, 95% CI = 1.00–1.01, *p* = 0.037). MELD score was correlated with hospital stay, 90-day mortality, and 4-year mortality in total PPH, and was associated with hospital stay and 4-year mortality in PPH without liver disease groups (hospital mortality for total PPH group, model 2: OR = 1.08, 95% CI = 1.02–1.15, *p* = 0.007; hospital mortality for PPH without liver disease group, model 1: OR = 1.07, 95% CI = 1.00–1.14, *p* = 0.045; 90-day mortality for total PPH group, model 2: OR = 1.06, 95% CI = 1.01–1.12, *p* = 0.017; 4-year mortality for total PPH group, model 1: OR = 1.06, 95% CI = 1.00–1.13, *p* = 0.048, model 2: OR = 1.08, 95% CI = 1.03–1.14, *p* = 0.003; 4-year mortality for PPH without liver disease group, model 2: OR = 1.08, 95% CI = 1.02–1.15, *p* = 0.011). ALBI score was correlated with hospital stay and 90-day mortality in total PPH, and was associated with 90-day mortality in PPH without liver disease groups (hospital mortality for total PPH group, model 2: OR = 2.54, 95% CI = 1.16–5.53, *p* = 0.019; 90-day mortality for total PPH group, model 1: OR = 2.45, 95% CI = 1.19–5.01, *p* = 0.014, model 2: OR = 2.69, 95% CI = 1.38–5.27, *p* = 0.004; 90-day mortality for PPH without liver disease group, model 1: OR = 2.31, 95% CI = 1.16–4.61, *p* = 0.017, model 2: OR = 2.97, 95% CI = 1.33–6.67, *p* = 0.008).

**TABLE 7:** Association between ALB, ALT, AST, and total bilirubin with prognosis of patients with PPH.

		Total PPH patients			Without liver disease		
Outcome		OR	95% CI	*P*-value	OR	95% CI	*P*-value
**Hospital mortality**							
ALB	Model 1	0.38	0.16–0.92	**0.027**	0.27	0.10–0.76	**0.015**
	Model 2	0.39	0.17–0.91	**0.028**	0.32	0.12–0.88	**0.027**
ALT	Model 1	1.00	1.00–1.01	0.139	1.00	1.00–1.01	0.950
	Model 2	1.00	1.00–1.01	**0.036**	1.00	1.00–1.01	0.985
AST	Model 1	1.00	1.00–1.01	**0.029**	1.00	1.00–1.01	0.142
	Model 2	1.00	1.00–1.01	**0.003**	1.00	0.99–1.01	0.166
Total bilirubin	Model 1	1.00	0.92–1.09	0.959	0.73	0.29–1.85	0.508
	Model 2	1.06	0.98–1.14	0.140	0.93	0.57–1.51	0.772
MELD	Model 1	1.04	0.97–1.13	0.220	1.07	1.00–1.14	**0.045**
	Model 2	1.08	1.02–1.15	**0.007**	1.04	0.96–1.13	0.353
ALBI	Model 1	1.87	0.78–4.45	0.159	1.87	0.81–4.33	0.143
	Model 2	2.54	1.16–5.53	**0.019**	2.47	0.93–6.51	0.068
**90-day mortality**							
ALB	Model 1	0.40	0.20–0.79	**0.008**	0.28	0.12–0.62	**0.002**
	Model 2	0.40	0.20–0.79	**0.009**	0.33	0.15–0.73	**0.006**
ALT	Model 1	1.00	1.00–1.01	0.231	1.00	1.00–1.01	0.695
	Model 2	1.00	1.00–1.01	0.106	1.00	1.00–1.01	0.482
AST	Model 1	1.00	1.00–1.01	0.130	1.00	1.00–1.01	0.266
	Model 2	1.00	1.00–1.01	**0.037**	1.00	1.00–1.01	0.238
Total bilirubin	Model 1	1.01	0.93–1.10	0.831	1.00	0.80–1.25	0.988
	Model 2	1.05	0.98–1.13	0.185	1.03	0.84–1.26	0.777
MELD	Model 1	1.04	0.98–1.11	0.237	1.06	1.00–1.12	0.061
	Model 2	1.06	1.01–1.12	**0.017**	1.04	0.97–1.11	0.309
ALBI	Model 1	2.45	1.19–5.01	**0.014**	2.31	1.16—-4.61	**0.017**
	Model 2	2.69	1.38–5.27	**0.004**	2.97	1.33–6.67	**0.008**
**4-year mortality**							
ALB	Model 1	0.83	0.44–1.59	0.583	0.73	0.36–1.46	0.370
	Model 2	0.86	0.46–1.60	0.633	0.79	0.40–1.59	0.520
ALT	Model 1	1.00	0.99–1.00	0.983	1.00	0.99–1.00	0.139
	Model 2	1.00	0.99–1.00	0.572	0.99	0.99–1.00	0.536
AST	Model 1	1.00	0.99–1.00	0.381	1.00	0.99–1.00	0.982
	Model 2	1.00	0.99–1.00	0.306	1.00	0.99–1.00	0.691
Total bilirubin	Model 1	1.04	0.93–1.15	0.526	1.19	0.83–1.73	0.344
	Model 2	1.03	0.95–1.13	0.435	1.29	0.79–2.10	0.315
MELD	Model 1	1.06	1.00–1.13	**0.048**	1.07	1.00–1.15	0.052
	Model 2	1.08	1.03–1.14	**0.003**	1.08	1.02–1.15	**0.011**
ALBI	Model 1	1.55	0.86–2.78	0.146	1.12	0.58–2.14	0.742
	Model 2	1.44	0.84–2.49	0.184	1.24	0.67–2.30	0.486

*For total patients with PPH, model 1 was adjusted for SOFA score, BUN, SpO_2_, liver disease, valvular disease and RR, and model 2 was adjusted for SAPS II score and valvular disease. For patients with PPH and without liver disease, model 1 was adjusted for SOFA score, BUN, SpO_2_, valvular disease, and MELD score, and model 2 was adjusted for SAPS II score and valvular disease. For 4-year mortality, only patients in the CareVue system who were followed up for at least 4 years were analyzed. The p-values of less than 0.05 are indicated in bold.*

### Predictive Ability of the Liver Function Index for the Prognosis of Patients With Primary Pulmonary Hypertension

**FIGURE 5:**
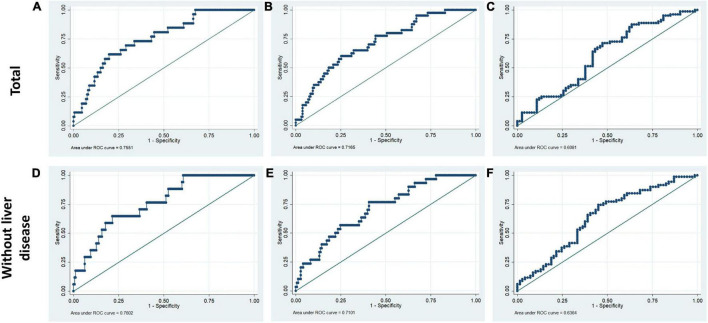
Receiver operating characteristic curves for liver function tests in hospital mortality, 90-day mortality, and 4-year mortality of patients with PPH. The ROC included ALB, ALT, AST, and total bilirubin. **(A,D)** Hospital mortality; **(B,E)** 90-day mortality; **(C,F)** 4-year mortality. **(A–C)** Total patients with PPH. **(D–F)** Patients with PPH and without liver disease.

The prediction model incorporating the total bilirubin, AST, ALB, and ALT was evaluated utilizing ROC curves for the purpose of determining the diagnostic significance. For hospital, 90-day and 4-year mortality, the diagnostic performance of liver function index was satisfactory (hospital mortality: AUC for total PPH group = 0.755, AUC for PPH without liver disease = 0.760; 90-day mortality: AUC for total PPH group = 0.717, AUC for PPH without liver disease = 0.710; 4-year mortality: AUC for total PPH group = 0.608, AUC for PPH without liver disease = 0.636, all *p* < 0.05) (Figure 5).

## Discussion

Within the human adult body, the liver is identified as the biggest solid organ, and it plays a crucial role in a variety of biological activities. The liver, lungs, and heart have a bidirectional and complicated interaction. Liver cirrhosis was linked to pulmonary arterial hypertension (portal hypertension) or aberrant pulmonary blood vessels dilation in the presence of severe hypoxia (hepatopulmonary syndrome) (15). In contrast, liver disease may be caused by heart and lung problems. Ischemic hepatitis and congestive liver disease are linked to cardiogenic shock and congestive heart failure, correspondingly. However, venous congestion caused by right ventricular malfunction could result in a significant elevation in hepatic blood volume, affecting liver function (16, 17). Unfortunately, there are no available outcome data for PPH patients without a history of hepatic failure or primary severe liver disease. Only ALB was correlated with the duration of ICU stay in the total PPH group, according to our findings. Abnormal ALB independently served as a risk variable for hospital mortality and 90-day mortality but not for 4-year mortality; however, it was significantly associated with 90-day and 4-year survival curves in both total PPH and PPH without liver disorders (Tables 2–7 and Figures 1, 3). Aberrant AST was linked to hospital mortality and 90-day survival curve in both total PPH and PPH without liver disease and independently served as risk variable for hospital and 90-day mortality only in total PPH group (Tables 2–7 and Figures 1, 3). ALT independently acted as a risk indicator for hospital mortality, whereas total bilirubin was only linked to hospital mortality only in the total group (Tables 6, 7). MELD score was an independent risk factor for hospital stay, 90-day mortality, and 4-year mortality in total PPH, and for hospital stay and 4-year mortality in PPH without liver disease groups. It was also significantly associated with 90-day and 4-year survival rates in total PPH and 4-year survival rates in PPH without liver disease (Tables 6, 7 and Figures 2, 4). ALBI score was an independent risk factor for hospital stay and 90-day mortality in total PPH, and for 90-day mortality in PPH without liver disease groups. It was also significantly associated with 90-day survival rates in both total PPH and PPH without liver disease (Tables 6, 7 and Figures 2, 4). The diagnostic performance of the predictive model combining the total bilirubin, AST, ALT, and ALB was moderately good for the hospital, 90-day, and 4-year mortality (Figure 5). In patients with PPH and with or without liver disorders, aberrant LFT was linked to short- and long-term patient prognosis, according to our findings.

Hypoalbuminemia may be a non-specific risk indicator for later-stage PAH since serum ALB plays a role in multiple physiological and pathological processes related to PAH progression, including hepatic dysfunction, starvation, and systemic inflammatory processes (18–21). Kawut et al. found that greater expression levels of ALB, as well as warfarin usage, acute vasoreactivity, and cardiac index, were independently linked to elevated transplant-free survival rates among patients with PAH (6). Snipelisky et al. showed that reduced serum albumin levels among patients with PAH are linked to elevated mortality rate and might function as an indicator of disease severity (7). Haddad et al. also showed a low level of serum ALB and elevated hospital mortality among patients with PAH representing a particularly high-risk cohort (22). Our research subject emphasized the analysis of patients with PPH and with and without liver disease, and our results were consistent with previous studies. Abnormal ALB independently functioned as a risk variable for hospital mortality and 90-day mortality and was significantly associated with 90-day and 4-year survival curves in both total PPH and PPH without liver disorders (Tables 2–7 and Figures 1, 3), suggesting that ALB may serve as an independent prognostic marker for PPH, irrespective of the presence or absence of liver disease in PPH patients. For patients with PPH and without liver disease, ALB was the main predictor, which may be a better surrogate for patient frailty and overall nutritional status and stage of RV dysfunction.

Hepatocellular injury pattern shaped by acute heart failure with reduced cardiac shock and output may cause a rapid increase in serum aminotransferase (AST/ALT) (23, 24). Meanwhile, patients with hepatic venous congestion caused by chronic heart failure are predominantly manifested by elevated cholestatic liver enzymes [hypoalbuminemia, gamma-glutamyltransferase (GGT), alkaline phosphatase (ALP), and bilirubin] rises (25, 26). Acute decompensated heart failure with reduced cardiac output causes a combined liver biochemical pattern with signals of hepatocyte damage (ALT and AST elevations) and cholestatic liver enzymes (ALP and bilirubin) elevations (23, 24). In the majority of research reports, bilirubin increase at baseline was linked to a worse outcome among patients with PAH. For PAH, increased total bilirubin levels were observed in 15–20% of patients (27). One further research discovered an increase in direct bilirubin level among 37% of patients and independently functioned as a risk indicator for mortality in individuals with idiopathic PAH (28). However, Hu et al. illustrated that total bilirubin was not correlated with the idiopathic PAH survival (10). This research illustrated that increased total bilirubin was correlated with hospital mortality for patients with PPH (Tables 3, 6). The inconsistency of the results may be due to the fact that this study included severe patients with pulmonary hypertension and right heart failure discharged from intensive care unit, while previous studies included mild and severe patients. In addition, the complexity of the disease may have resulted in inconsistent results across the included populations. Moreover, several smaller studies showed mildly elevated total bilirubin, ALP, and GGT for patients with PAH, while elevation in AST/ALT was less common (6, 8, 22, 29, 30). However, our results showed that aberrant AST was linked to hospital mortality and 90-day survival curve in both total PPH and PPH without liver disease, while served as a risk variable for hospital and 90-day mortality in an independent manner only in the total PPH group (Tables 2–7 and Figures 1, 3). ALT independently functioned as a risk variable for hospital mortality was associated with hospital mortality only in the total group (Tables 6, 7). These results suggested AST but not ALT was more sensitive for predicting mortality in patients with PPH and without liver disorders, and it could be involved in the PPH process. However, when we considered these four indicators in combination, rather than analyzing them individually, we found that the model composed of these four indicators had an outstanding prognostic predictive ability for mortality in patients with PPH irrespective of the presence or absence of liver disease (Figure 5). Taken together, LFTs might be a viable prognostic indicator for PPH.

## Limitations

This study has some limitations. First, this was a single-center retrospective non-randomized study. Second, the therapy of pulmonary hypertension has drastically changed during the last decade. More recent data should be analyzed for more precise conclusion. Moreover, some PH drugs affect liver function; however, drug information for primary pulmonary arterial hypertension in the database was seriously missing. Therefore, we had to abandon the analysis in this study. Furthermore, the sample size of the study was small, especially the low number of non-survivors. More robust studies with larger sample size and meta-analyses should be done to further illustrate the relationship between LTFs and pulmonary hypertension. Then, the disease definition of the MIMIC III database is based on the ICD-9-CM code. Therefore, some important information was lacking because of the limitations of the database itself, such as echocardiography results, electrocardiogram results, pulmonary artery pressure, and risk scores. These were the limitations of the database itself.

## Conclusion

In this research, we discovered that aberrant ALB was associated with longer ICU stay among critically ill patients with PPH. Among patients with PPH and with or without liver illness, aberrant LFTs were linked to increased hospital mortality, 90-day mortality, and 4-year mortality. The model composed of LFTs had a good prognostic predictive ability for mortality in critically ill patients with PPH irrespective of the presence or absence of liver disease.

## Data Availability Statement

The raw data supporting the conclusions of this article will be made available by the authors, without undue reservation, to any qualified researcher.

## Ethics Statement

The studies involving human participants were reviewed and approved by the institutional review boards of the MIT (Cambridge, MA, United States) and BIDMC (Boston, MA, United States). Written informed consent for participation was not required for this study in accordance with the national legislation and the institutional requirements.

## Author Contributions

JH and BH conceived and designed the study. DW, SH, GX, SW, ZL, and LX provided, selected, assembled, analyzed, and interpreted the data. All authors contributed to data analysis, drafting and critically revising the article, and agreed to be accountable for all aspects of the work. All authors have read and confirmed that they met ICMJE criteria for authorship.

## Conflict of Interest

The authors declare that the research was conducted in the absence of any commercial or financial relationships that could be construed as a potential conflict of interest.

## Publisher’s Note

All claims expressed in this article are solely those of the authors and do not necessarily represent those of their affiliated organizations, or those of the publisher, the editors and the reviewers. Any product that may be evaluated in this article, or claim that may be made by its manufacturer, is not guaranteed or endorsed by the publisher.
